# Mechanical Compression Regulates Brain Cancer Cell Migration Through MEK1/Erk1 Pathway Activation and GDF15 Expression

**DOI:** 10.3389/fonc.2019.00992

**Published:** 2019-09-27

**Authors:** Maria Kalli, Chrysovalantis Voutouri, Angeliki Minia, Vaia Pliaka, Christos Fotis, Leonidas G. Alexopoulos, Triantafyllos Stylianopoulos

**Affiliations:** ^1^Cancer Biophysics Laboratory, Department of Mechanical and Manufacturing Engineering, University of Cyprus, Nicosia, Cyprus; ^2^ProtATonce Ltd, Athens, Greece; ^3^Department of Mechanical Engineering, National Technical University of Athens, Athens, Greece

**Keywords:** GBM, midline shift, metastasis, small GTPases, phosphoproteomic screen, c-Jun, Akt

## Abstract

Mechanical compression is a common abnormality of brain tumors that has been shown to be responsible for the severe neurological defects of brain cancer patients representing a negative prognostic factor. Indeed, it is of note that patients that undergo resection exhibited higher survival rates than those subjected to biopsy only, suggesting that compressive forces generated during brain tumor growth play a key role in tumor progression. Despite the importance of mechanical compression in brain tumors, there is a lack of studies examining its direct effects on brain cancer cells and the mechanisms involved. In the present study, we used two brain cancer cell lines with distinct metastatic potential, the less aggressive H4 and the highly aggressive A172 cell lines, in order to study the effect of compression on their proliferative and migratory ability. Specifically, we used multicellular tumor spheroids (MCS) embedded in agarose matrix to show that compression strongly impaired their growth. Using mathematical modeling, we estimated the levels of compressive stress generated during the growth of brain MCS and then we applied the respective stress levels on brain cancer cell monolayers using our previously established transmembrane pressure device. By performing a scratch assay, we found that compression strongly induced the migration of the less aggressive H4 cells, while a less pronounced effect was observed for A172 cells. Analysis of the gene expression profile of both cell lines revealed that GDF15 and small GTPases are strongly regulated by mechanical compression, while GDF15 was further shown to be necessary for cells to migrate under compression. Through a phospho-proteomic screening, we further found that compressive stimulus is transmitted through the MEK1/Erk1 signaling pathway, which is also necessary for the migration of brain cancer cells. Finally, our results gave the first indication that GDF15 could regulate and being regulated by MEK1/Erk1 signaling pathway in order to facilitate the compression-induced brain cancer cell migration, rendering them along with small GTPases as potential targets for future anti-metastatic therapeutic innovations to treat brain tumors.

## Introduction

Glioma is the most common primary malignant brain tumor and arises from glial cells, a type of supporting tissue for the brain cells. A glioma can be differentiated into low grade or benign glioma (grade I–III) and a high grade or malignant glioma, such as glioblastoma (Grade IV), also termed Glioblastoma multiforme (GBM), that is found to be highly cancerous containing a very high blood supply and comprising a necrotic and cystic tissue (1, 2).

A common characteristic and a major cause of the clinical symptoms seen in patients suffering from brain cancer, is the compression of brain tissue by the primary tumor mass. As the tumor grows in the cranium, it must displace the surrounding tissue and this tumor growth-induced deformation of the brain can cause severe disabilities to patients, representing a negative prognostic factor (3). Specifically, shift of the cranial midline is a common characteristic between patients diagnosed with a malignant glioma (GBM), while patients with midline shift tend to acquire significant brain compression, which is linked to rapidly developing and strong neurologic deficiencies (4). Moreover, it has been previously found that midline shift is adversely correlated with the survival of patients that were able to subsist a biopsy, but not in the patients that were able to undergo resection (4), suggesting that resection and the subsequent alleviation of compressive forces generated by tumor growth could improve treatment (4, 5). However, despite the importance of mechanical compression in brain tumors and while its effect in other tumor types is actively being studied (6–10), there is no study examining its direct effects on brain cancer cells and the mechanisms involved.

It is well-established that in gliomas there is often an overexpression of the Epidermal Growth Factor Receptor (EGFR) and a downregulation or mutations of the tumor suppressor TP53 transcription factor (11). EGFR can regulate a variety of signaling pathways including Ras/Raf/MEK/Erk, PI3K/Akt/mTOR, or Jak/STAT that are all implicated in cell survival, proliferation and migration (12). Indeed, Ras guanosine triphosphate (Ras-GTP) has been documented in cell lines and primary brain tumors, and it is able to translate extrinsic signals into the Raf-MEK-Erk, or into either the PI3K-PKB or the PI3K-Rac-Rho pathways, which influence cell survival and migration (11–13). Notably, almost all GBMs show increased activity of PI3K pathway, while its negative regulator PTEN, that is implicated in survival, proliferation and migration, is usually downregulated (12). Moreover, the signaling pathway regulated by cytosolic tyrosine kinase Janus kinase (JAK)-family proteins and transcription factor signal transducer and activator of transcription (STAT)-family proteins is efficiently activated, especially downstream of cytokine receptors. Based on these data, we expect that mechanical compression could alter a combination of these pathways in order to regulate cellular responses, such as gene expression, cell proliferation, and migration. In fact, through a signal transduction mechanism, mechanical compression could regulate the expression of several genes that are known to be responsive to morphological and cytoskeletal changes, such as the small GTPases and the Growth Differentiation Factor-15 (GDF15) (14–17). GDF15 belongs to the Transforming Growth Factor β (TGFβ) superfamily of cytokines, and it is found to be elevated in the serum of patients with high grade gliomas representing a negative prognostic factor (18). Moreover, this molecule has attracted much attention because of its pleiotropic functions in tumor progression, acting either as a tumor suppressor at the early stages of a tumor, and as a tumor promoter in the late stages (19–21). Indeed, it has been shown that GDF15 exhibited higher expression levels in secondary and lower expression in primary brain tumors, while its role in brain tumor progression was found to be inconsistent acting in a cell type- and context-dependent manner (18, 19, 21–23).

In the present study, we used the non-metastatic glioma H4 cell line and the highly malignant and metastatic GBM cell line A172, in order to investigate how brain tumor cells respond to mechanical compression by examining their proliferative and migratory abilities. Given the fact that there is a limited number of studies estimating the levels of the compressive stress in brain tumors (3, 24), we employed mathematical modeling to calculate *in vitro* the magnitude of stress developed during the growth of Multicellular Spheroids (MCS) embedded in an agarose matrix. The estimation of compressive stress levels enabled us to apply a controlled and predefined mechanical compression on cell monolayers to investigate the mechanism by which it regulates gene expression and cellular behavior. Through a phospho-proteomic screening, we set out to identify a possible molecular mechanism by which mechanical compression can regulate brain cellular responses, similarly to the mechanism identified for pancreatic cancer cells (10). The identification of the compression-induced signal transduction mechanisms could suggest novel therapeutic targets for the treatment of patients with brain tumors, further enhancing the importance of targeting the compression-induced tumor progression.

## Materials and Methods

### Cell Culture

Brain neuroglioma (H4) and glioblastoma (A712) cell lines, were purchased from American Type Culture Collection (ATCC) and were cultured in Dulbecco's Modified Eagle's Medium (DMEM) containing 10% Fetal Bovine Serum (FBS) and 1% antibiotics. Cells were incubated in a humidified incubator at 37°C and 5% CO_2_.

### Multicellular Spheroid (MCS) Formation

H4 and A172 MCS were formed using the “hanging drop” technique (25–27). Briefly, cells were counted and then put in suspension at a final concentration of 2.0–2.5 × 10^4^ cells/ml. Next, around 500 cells were placed on the inside of the cover of a 100-mm culture dish as hanging drops (20 μl) and were left for 48 h. The formed spheroids were transferred into a 96-well plate, which was pre-coated with 50 μl of 1% low-melting agarose (concentration was obtained by mixing stock solution of 4% agarose in DMEM). Culture medium, for free spheroids, or 1% low-melting agarose was then added, and pictures were taken after 24 h using a Nikon Eclipse optical microscope. Spheroids were incubated at 37°C for a total period of 21 days and pictures were taken every 2–3 days. Spheroid size (area) was measured using the ImageJ software and difference in spheroids' size was compared to the initial size at Day 1 according to the following formula:

((Spheroid size at Day 21 – Spheroid size at Day 1)/(Spheroid size at Day 1)) × 100.

### Estimation of Compressive Stress Level *in vitro*

To estimate the compressive stress level, we used a previously described method (28). Specifically, to mathematically model the growth and mechanical behavior of tumor spheroids within an agarose matrix, a finite elements model was constructed and the growth of the spheroid within the agarose was simulated. To simulate spheroid growth, the multiplicative decomposition of the deformation gradient tensor, **F**, was employed (29), a methodology that has been applied successfully to model the growth of solid tumors (30–32) as well as other soft tissues (33, 34). The model considered only the solid phase of the spheroid and accounted for its growth and mechanical interactions with the surrounding agarose matrix. Therefore, **F** was divided into two components:

(1) $$F=F_{\text{e}}F_{\text{g}}\;,$$

where **F**_**e**_ is the elastic component of the deformation gradient tensor that accounts for mechanical interactions with the surrounding matrix or with any other external structure and **F**_**g**_ is the component that accounts for spheroid growth.

The growth of the tumor spheroid was considered to be isotropic and therefore, the **F**_**g**_ was given by:

(2) $$\begin{array}{r} {\text{F}}_{g}=\lambda_{g}\text{I} \end{array}$$

where λ_*g*_ is the growth stretch ratio, which was described by (32):

(3) $$\begin{array}{r} \frac{d\lambda_{g}}{dt}=-a\;, \end{array}$$

where *t* is the time and α describes the spheroid growth rate, the value of which was estimated experimentally for each cell line by measuring the growth of the spheroids.

Finally, to describe the elastic response of both the MCS spheroids and the agarose matrix, we employed the constitutive equation for the compressible neo-Hookean material (35). The strain energy density function, *W*, of the neo-Hookean equation is:

(4) $$\begin{array}{r} W=0.5\mu \left(-3+{\text{II}}_{1}\right)+0.5\kappa {\left(-1+J_{e}\right)}^{2}, \end{array}$$

where μ is the shear modulus and κ is the bulk modulus *J*_*e*_ is the determinant of the elastic deformation gradient tensor **F**_**e**_ and $II_{1}=I_{1}J_{e}^{-2/3}$. I_1_, I_2_, and I_3_ are the invariants of the right Cauchy-Green deformation tensor, which is evaluated from the elastic part of the deformation gradient tensor, **F**_**e**_.

The Cauchy stress tensor, **σ**, was calculated by the strain energy density function as (36):

(5) $$\begin{array}{r} \sigma =J_{e}^{-1}{\text{F}}_{e}\frac{\partial W}{\partial {\text{F}}_{e}^{T}} \end{array}$$

Finally, the linear momentum balance was solved assuming a quasi-static problem in the absence of body forces:

(6) $$\begin{array}{r} \nabla \cdot \sigma =0 \end{array}$$

A model was constructed in the commercial finite elements software COMSOL v. 5.2 to solve the system of Equations (1)–(6). The growth of spherical MCS within an agarose matrix of cubic shape was modeled and because of symmetry, the one eighth of the domain was solved, by applying a symmetry boundary condition at the symmetric boundaries and a stress-free condition at the free surfaces (Supplementary Figure 1B) (28). The mechanical properties of the agarose were measured with mechanical testing, while the properties of the MCS were assumed to be 15.2 kPa for H4 cells and 9.6 kPa for A172 cells according to recently published data (37).

### Material Properties of the Agarose Matrix

In order to estimate the material properties of the agarose gel (i.e., elastic modulus) stress-strain experiments were performed (28). Unconfined compression, stress-strain experiments were carried out using a commercial high precision mechanical testing system (Instron 5944, Norwood, MA, USA). Samples of 1% agarose gel were cut with approximate dimensions 3 × 3 × 2 mm (length × width × thickness). According to the stress-strain protocol the specimens were placed between two parallel platens and they were compressed to a final strain of 15% with a strain rate of 0.05 mm/min, the minimum rate the system can apply in order to avoid any transient, poroelastic effects. Stress was calculated as the force measured on the load cell divided by the initial surface area of the specimen (i.e., 1st Piola-Kirchhoff stress), and displacement data were converted to strain as ε = Δ*l/l*_0_, where Δ*l* is the change in the length of the specimen in the direction of compression and *l*_0_ the initial, undeformed length. The elastic modulus was calculated from the slope of the stress-strain curve at 15% strain.

### *In vitro* Compression of Cell Monolayers

In order to apply mechanical compression on brain cancer cell monolayers, a previously published procedure was followed (9, 10). Briefly, cells were grown to form a monolayer on a transwell insert and then an agarose cushion was placed on the top of the cells. A piston was then placed to apply 2.0 and/or 4.0 mmHg for 16 h (Supplementary Figure 2). These values of mechanical compression were within the range that were computed by the mathematical model. Control cells were covered with an agarose cushion only.

### *In vitro* Wound Healing Assay

A scratch wound assay was performed as previously described (9, 10). In brief, a wound was created on H4 and A172 cell monolayers by a 200 μL pipette tip and then cells were washed twice with Phosphate Buffered Saline (PBS) and compressed as indicated in each figure legend for 16 h. Images from at least three distinct fields from each condition were taken at 0 and 16 h. ImageJ software was used to calculate the wound area and then the wound closure was quantified according to the following equation:

((Wound area at 0 hours – Wound area at 16 hours)/(Wound area at 0 hours)) × 100.

### Cell Viability Assay

Cell viability of cancer cells, indicative of the total cell number, was estimated using Alamar Blue reagent (Invitrogen Life Technologies) before and after the application of compression following the manufacturer's guidilines.

### Gene Expression Analysis

Total RNA extraction from brain cancer cells, RNA purification, cDNA synthesis and gene expression analyses at the mRNA level were performed as described previously (9, 10). All primers used are shown in Supplementary Table 1. Relative gene expression at the mRNA level was quantified using the ΔΔCt method where uncompressed and untreated cells were used as calibrators.

### Immunoblotting

For protein expression analysis, a standard immunoblotting protocol was followed as described previously (10). The antibodies used in this study were: anti-GDF15 (Santa Cruz Biotechnology), anti-RhoB total (Abcam), anti-phospho (Thr202/Tyr204) Erk1/2 (Cell Signaling Technology), anti-total Erk1/2 (Cell Signaling Technology), anti-phospho-c-Jun (S63, Abcam), and anti-total-c-Jun (Abcam). Antibodies against β-actin or β-tubulin were used as loading control.

### siRNA Transfections

H4 and A172 cells were transfected with 100 nM non-specific control siRNA (siCTRL) or siRNA against GDF15 or RhoB (Santa Cruz Biotechnology) using Lipofectamine 2000 reagent (Invitrogen Life Technologies). Cells were then compressed by 4.0 mmHg and a scratch wound assay was performed. Cells were harvested 64 h post-transfection and silencing efficiency was verified by Western Blotting and Real Time PCR.

### Cell Treatments With MEK1 Inhibitor

To study the role of MEK1 pathway in brain cancer cell migration under compression, a MEK1 inhibitor (PD98059, MedChemExpress) was selected. Cells were cultured in transwell inserts with low serum medium for 24 h and were then pre-treated with 20 μM PD98059 or equivalent volume of DMSO for 60 min. The manufacturer's guidilines were followed for the selection of the proper PD98059 concentration. Cells were then subjected to a scratch assay in the presence of mechanical compression (4.0 mmHg).

### Sample Preparation and Phosphoprotein's Measurements

Total protein extracts were isolated from control and compressed brain cancer cells from 3 biological replicates using radio immunoprecipitation assay (RIPA) buffer which contained a protease inhibitor cocktail tablet (Sigma). Protein concentration was determined by the BCA protein assay kit (Pierce) and finally, 200 μg/ml of each cell lysate were subjected to phosphoprotein measurements. 18 custom dual-antibody Luminex assays were developed using ProtATonce (Athens, Greece) multiplex assay service as described previously (10). The exact protocol, the phospho-proteins tested, and the normalization procedure can be found in detail in Supplementary Material.

### Statistics

The experimental data are presented as means with standard errors (SEM). Statistical significances were examined by Student's *t*-test using two-tail distribution or ANOVA paired analysis using the software program GraphPadPrism (6.0 for Windows; GraphPad Prism Software Inc., San Diego, CA). Differences with *p* < 0.05 were considered as significantly different and are nominated with an asterisk in each figure (^*^).

## Results

### The Growth of Brain Cancer MCS Is Hindered by the Surrounding Agarose Matrix

**Figure 1:**
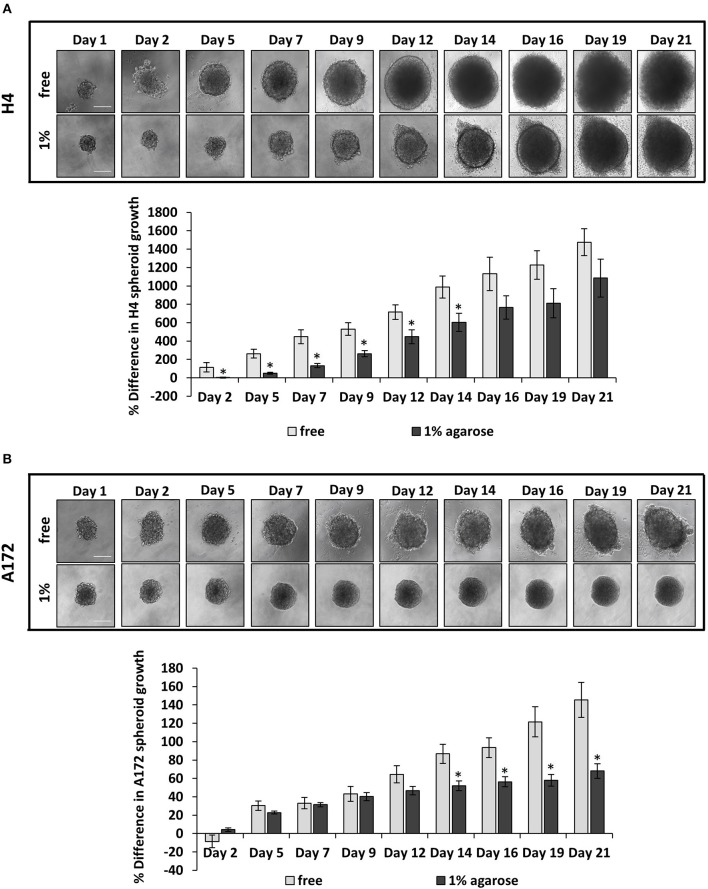
The growth of brain cancer MCS is hindered by a surrounding agarose matrix. **(A,B)** Multicellular spheroids (MCS) composed by H4 **(A)** or A172 **(B)** cells, were embedded in 1% agarose matrix or in free suspension and grew for 21 days. Images were taken every 2–3 days with an optical microscope and the area of each spheroid was quantified using ImageJ. The average % difference in each spheroid size was calculated and plotted for each cell line ±SE (≥6 spheroids/condition; 2 independent experiments; number of total replicates *n* = 12–18). Error bars indicate standard error (SEM). Asterisks (*) indicate a statistically significant difference between free spheroids and spheroids embedded in 1% agarose matrix. Scale bar: 0. 15 mm.

Several studies have dealt with the effect of mechanical compression on tumor growth *in vitro*, by embedding tumor spheroids in agarose matrices of varying concentrations (6, 38–40). It has been established that tumor growth is impaired by the surrounding matrix and this hindrance depends on the matrix concentration. However, there is a limited number of studies showing the effect of the surrounding matrix on brain tumor growth (41). To this end, to confirm the effect of matrix compression on brain tumor growth *in vitro*, MCS composed of H4 or A172 brain cancer cells were formed and embedded in 1% agarose matrix or grown in free- suspension for 21 days. We found that the presence of agarose strongly impaired the growth of brain MCS, as similarly observed for collagen matrices (41), with H4 spheroids presenting a delay of growth compared to the respective control spheroids, and A172 spheroids exhibiting a complete cease of their growth as compared to the free spheroids (Figures 1A,B). Our results agree with studies employing colon and breast cancer cells (6, 38, 40) and data from *in vivo* measurements of brain tissue pressure in patients or animal models with brain tumors (24, 42).

### Estimation of Compressive Stress Generated During the Growth of Brain Cancer Mcs in Agarose Matrix

**Figure 2:**
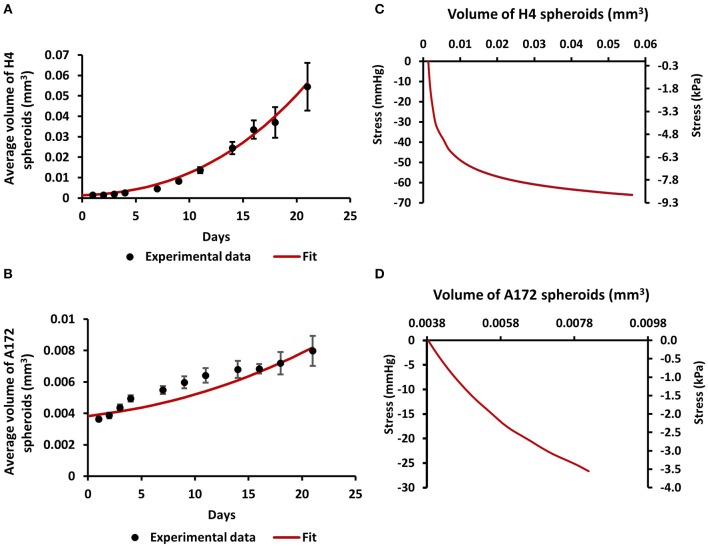
Estimation of compressive stress generated during the growth of brain cancer MCS within an agarose matrix. **(A,B)** Fit of the mathematical model to the experimental data of the growth of H4 **(A)** and A172 **(B)** spheroids. **(C,D)** The calculated by the model bulk stress generated during the growth of H4 **(C)** and A172 **(D)** spheroids.

In order for the MCS to grow in size, they should displace the surrounding matrix similar to the physiological growth of tumors (Figure 1). To estimate the compressive stress that is exerted from the agarose on the spheroids to resist their expansion, we employed mechanical testing experiments and mathematical modeling. We first measured the elastic modulus of the agarose gel in unconfined compression and found it to be equal to 75 ± 3.75 mmHg (10.47 ± 0.5 kPa). Subsequently, we employed the neo-Hookean constitutive equation to fit the experimentally derived stress-strain results and to derive the value of the shear modulus μ and bulk modulus κ in (Equation 4) assuming a Poisson's ratio of 0.2 (Supplementary Figure 1A). Then, the growth of the MCS was simulated and the predicted by the mathematical model growth curves were fitted to the experimental data (Figures 2A,B) by varying the parameter α in Equation 3 and the developed mechanical stress was calculated from the model (Figures 2C,D). Figures 2C,D presents the bulk stress at the center of the MCS, calculated as the average of the diagonal components of the Cauchy stress tensor (radial σ_*rr*_ and circumferential σ_θθ_, σ_ϕϕ_), i.e., $\bar{\sigma }=\left(\sigma_{rr}+\sigma_{\theta \theta }+\sigma_{\phi \phi }\right)/3$. The compressive stress was calculated in the range of 0–60 mmHg (0.0–8 kPa). In particular, the level of stress developed during the growth of H4 spheroids (0–60 mmHg/ 0.0–8 kPa) was much higher than that of A172 spheroids (0–26 mmHg/0.0–3.5 kPa), which would be expected as the H4 spheroids grew to a much higher volume than that of A172. The estimated levels of stress in both cell lines were in agreement with previous *in situ* (2.8–60.1 mmHg/0.37–8.0 kPa), *in vivo* (4–28 mmHg/0.53–3.53 kPa), and *in vitro* estimations (28–120 mmHg/3.7–16.0 mmHg) (6, 24, 38, 42, 43) of intratumoral compressive stress.

### Mechanical Compression Differentially Regulates the Migration of Brain Cancer Cells

**Figure 3:**
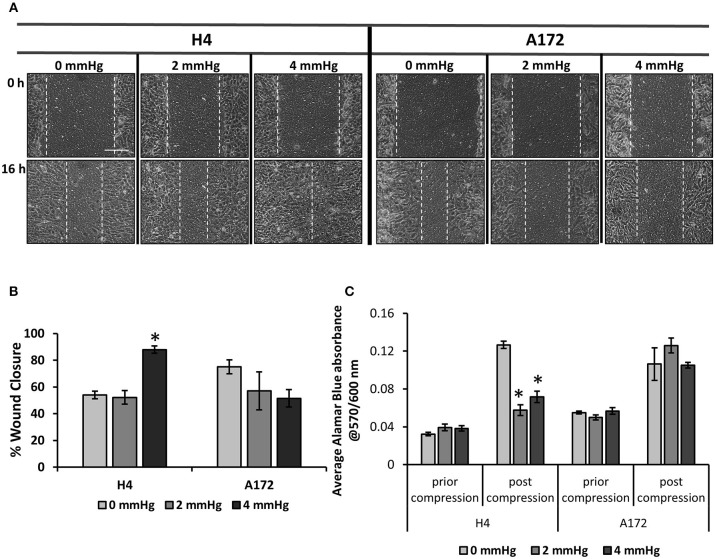
Mechanical compression differentially regulates the migration of brain cancer cells. **(A)** Brain cancer cells, H4 (left) and A172 (right) were grown in transwell inserts to form a monolayer. A scratch wound was then introduced and compression (0, 2.0, and 4.0 mmHg) was applied for 16 h. Pictures from at least 3 different fields per condition were taken with an optical microscope (10× magnification) prior and post compression. Scale bar: 0.15 mm. **(B)** Cell-free area was quantified using ImageJ software and the average percentage of wound closure from was plotted for each cell line (≥3 different fields/condition; ≥2 independent experiments; number of total replicates *n* = 6–9). **(C)** Brain cancer cells lines were counted and seeded with equal density in 6-well transwell inserts. Alamar Blue was added in culture medium (10%) and absorbance was measured prior- and post- compression at 570/600 nm. Absorbance of Alamar Blue is indicative of the total cell number. The asterisk denotes a statistically significant difference compared to the uncompressed condition.

To determine how mechanical compression affects the cellular behavior of brain cancer cells, we used our previously described transmembrane pressure device (9, 10) in order to compress cell monolayers (Supplementary Figure 2). Based on the calculations of compressive stress, we employed 2.0 and 4.0 mmHg (0.26 and 0.53 kPa) of stress, as these levels can be generated in the first 2 days of spheroid's growth in the agarose matrix and are supposed to be an early-transmitted stress stimulus in brain cancer cells, at least *in vitro*. We found that while the 2.0 mmHg stress did not cause any significant change in the migratory ability of brain cancer cells, the 4.0 mmHg stress level was able to greatly enhance the migration of glioma H4 cells. Regarding the A172 cells, mechanical compression was shown to have no effect or slightly impair the migratory ability of GBM A172 cells, without any statistically significant difference compared to uncompressed cells (Figures 3A,B). Moreover, we observed that mechanical compression strongly reduced the cell number of H4 cells without any effect on A172 cells, at least until ~24 h post-compression, as indicated by Alamar Blue assay (Figure 3C, Supplementary Figure 3). These results further support our hypothesis that the higher wound closure of compressed H4 cells was not due to higher cell proliferation rate but because of a compression-induced migratory effect.

### Mechanical Compression Regulates the Expression of GDF15 and Small GTPases in Brain Cancer Cells

**Figure 4:**
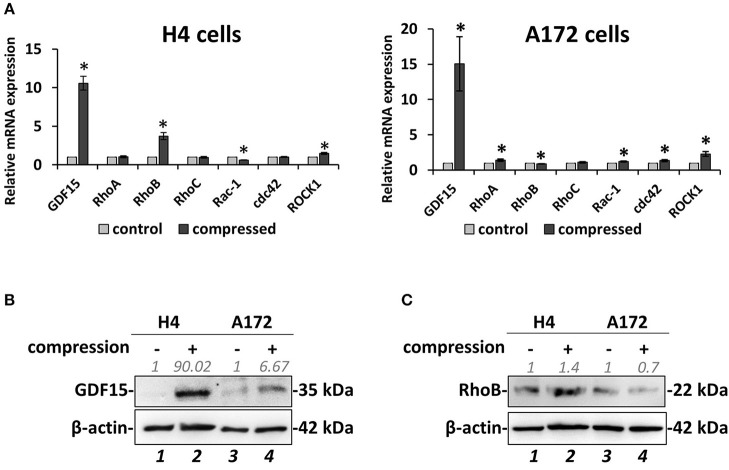
Mechanical compression induces the expression of GDF15 and regulates the expression of small GTPases. **(A)** Brain cancer cells, H4 (left) and A172 (right) were subjected to 4.0 mmHg of compressive stress for 16 h and the expression of migration-related genes was measured by qPCR. The mRNA expression in each sample was quantified by the ΔΔCt method using the expression in uncompressed cells as a reference. Bar graphs represent the mean fold change ±SE of three biological replicates (number of total replicates *n* = 9). Statistically significant changes between compressed and uncompressed cells are indicated by an asterisk (*) (*p* < 0.05). **(B,C)** Representative Western blotting showing the expression of GDF15 **(B)** and RhoB **(C)** in the compressed H4 and A172 cells. B-actin was used to verify equal protein loading. Protein expression was quantified using ImageJ software and the fold change is indicated in gray font.

Based on the data described above suggesting that compressive stress can differentially regulate the migration and growth of brain cancer cells, we proceeded to analyze the gene expression profile of both cell lines in order to molecularly explain the migratory effect that was observed in response to compression. To this end, we compressed H4 and A172 cells at 4.0 mmHg, as this level of compression caused significant changes in the migration of both cell lines, and we then examined the expression of GDF15, which is a stress sensor and was found to be consistently upregulated as a response to compression (9, 10). Moreover, we analyzed the expression of several small GTPases, such as Ras Homolog family members (RHO) A-C and Cell division cycle 42 (cdc42), Rac family small GTPase 1 (Rac-1), and Rho associated coiled-coil containing protein kinase (ROCK1), which are all directly linked to actin cytoskeleton organization, being implicated in mechano-transduction, cell adhesion and migration (14). Real-time PCR and Western Blotting revealed that *GDF15* exhibited a dramatic increase in both cell lines at both the mRNA and protein level (Figures 4A,B), while *RhoB GTPase* exhibited the most dramatic change among all small GTPases tested, showing an increase in H4 cells and a slight decrease in A172 cells (Figures 4A–C). Notably, the rest of the genes showed negligible changes as compared to the changes showed for *RhoB* and *GDF15*, with *RhoA* and *Rac1* exhibiting a slight increase in compressed A172 cells, *cdc42* showing a small decrease in H4 and an increase in A172, while *ROCK1* showed an increase in both cell lines. The expression of GDF15 and RhoB GTPase has been previously shown to be upregulated in response to compression (10), and to have increased levels in high grade gliomas rendering them as potential targets for the treatment of this type of cancer (16, 19, 44). Therefore, our results allowed us to form the hypothesis that these genes are necessary for compression-induced brain cancer cell migration, and thus we further proceeded to examine their role in this process.

### Silencing of RhoB GTPase Does Not Affect the Migration of Brain Cancer Cells Under Mechanical Compression

**Figure 5:**
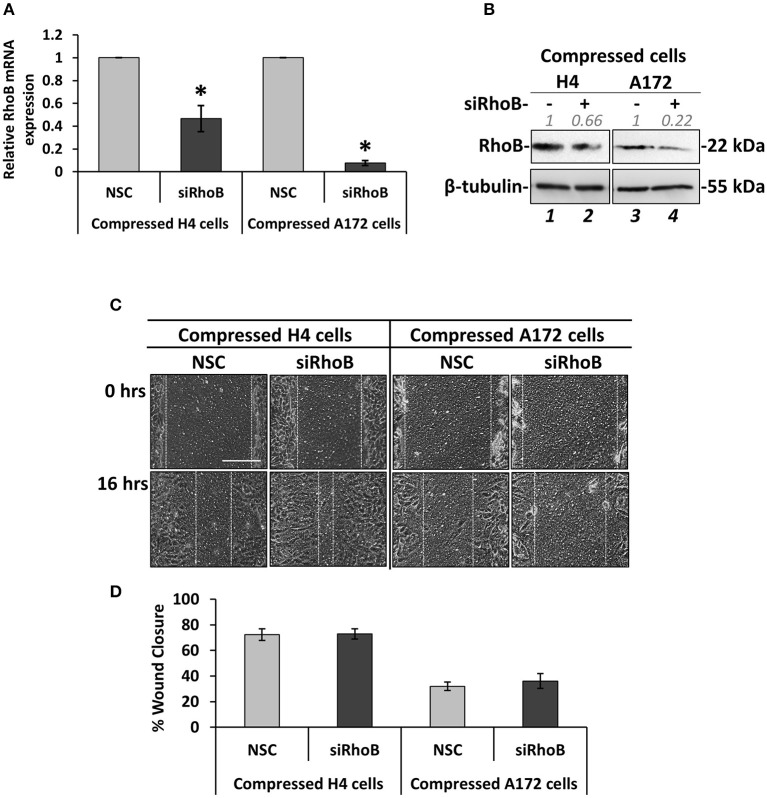
Silencing of RhoB GTPase does not affect the migration of brain cancer cells under mechanical compression. **(A)** H4 and A172 brain cancer cells were transiently transfected with siRNA against RhoB and were compressed by 4.0 mmHg in 2% FBS containing DMEM. Total RNA was then isolated and *RhoB* mRNA expression was quantified by qPCR. Each bar indicates the mean fold change ±SE of three independent experiments (number of total replicates *n* = 9). Asterisk (*) indicates a statistically significant difference (*p* < 0.05). **(B)** Western Blotting showing that RhoB protein expression has been successfully reduced in compressed siRhoB-treated cells (lanes 2 and 4) compared to compressed control cells (lanes 1 and 4). Protein expression was quantified using ImageJ software and the fold change is indicated in gray font. **(C)** H4 and A172 cells knockdown for siRhoB were compressed by 4.0 mmHg in low-serum medium and then subjected to a scratch wound healing assay for 16 h. Control cells were treated with non-specific control siRNA (NSC). Scale bar: 0.2 mm. **(D)** Graph showing the percentage wound closure as quantified using ImageJ software (≥3 different fields/condition; ≥3 independent experiments number of total replicates *n* ≥ 10). No statistically significant differences in wound closure were observed between both H4 and A172 siRhoB-treated cells and the respective NSC-treated cells.

Based on the fact that mechanical compression upregulates the expression of RhoB GTPase in H4 cells, and in order to identify how it could be implicated in the stress-induced brain cancer cell migration, it was transiently silenced using a siRNA-mediated silencing approach. Mechanical compression was then applied for 16 h. As shown in Figure 5, *RhoB* was effectively depleted in both the H4 and A172 cells following siRNA treatment, as revealed by qPCR and Western Blotting (Figures 5A,B). Regarding cell migration, our results showed that RhoB silencing did not affect the migratory ability of compressed or uncompressed H4 and A172 cells (Figures 5C,D, Supplementary Figures 4A,B), indicating that other members of the small GTPases family, such as RhoA, RhoC, or Rac-1 could possibly have a role in rescuing the absence of RhoB GTPase in both cell lines.

### GDF15 Expression Is Necessary for Mechanical Compression-Induced Brain Cancer Cell Migration

**Figure 6:**
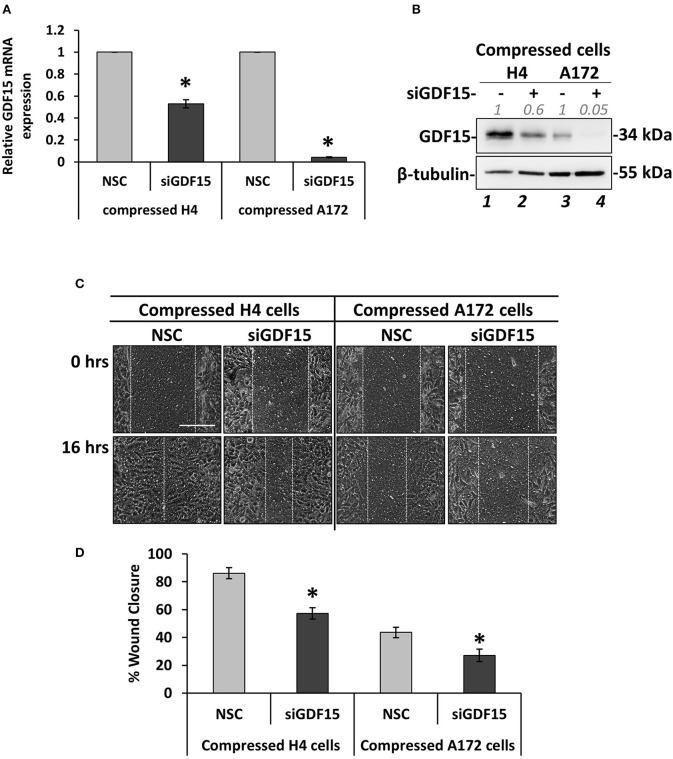
GDF15 expression is necessary for mechanical compression-induced brain cancer cell migration. **(A)** H4 and A172 brain cancer cells were treated with siRNA against GDF15 and were compressed by 4.0 mmHg in 2% FBS containing DMEM following by total RNA and protein isolation. *GDF15* mRNA expression was quantified by qPCR. Each bar indicates the mean fold change ±SE of three independent experiments (number of total replicates *n* = 9). Asterisk (*) indicates a statistically significant difference (*p* < 0.05). **(B)** Western Blotting showing that GDF15 protein expression has been successfully reduced in compressed siGDF15-treated cells (lane 2 and 4) compared to compressed control cells (lane 1 and 4). Protein expression was quantified using ImageJ software and the fold change is indicated in gray font. **(C)** GDF15 knockdown H4 and A172 cells were compressed by 4.0 mmHg in low-serum medium and then subjected to a scratch wound healing assay for 16 h. Control cells were treated with non-specific control siRNA (NSC). Scale bar: 0.2 mm. **(D)** Graph showing the percentage wound closure as quantified using ImageJ software. Statistically significant difference in wound closure of siGDF15-treated H4 and A172 cells compared to NSC siRNA-treated cells is indicated with an asterisk (*) (≥3 different fields/condition; 4 independent experiments; number of total replicates *n* ≥ 12; *p* < 0.05).

According to Figure 4, GDF15 showed a dramatic elevation in both compressed H4 and A172 cells, which is in accordance with our previous studies that employed pancreatic tumor cells to show that this gene is consistently upregulated as a response to compression (9, 10). To this end, we set to examine whether GDF15 is also necessary for brain cancer cell migration under stress conditions by transiently transfect cells with siRNA against GDF15 (siGDF15). Mechanical compression was then applied for 16 h. As shown in Figure 6, *GDF15* was reduced both at the mRNA and protein level following siGDF15 treatment (Figures 6A,B). Regarding the metastatic potential of siGDF15-treated cells, a scratch assay revealed that the migratory ability of both brain cancer cell lines was impaired when GDF15 was reduced in the presence of mechanical compression (Figures 6C,D), while in uncompressed conditions *GDF15* silencing impaired only the migration of A172 cells (Supplementary Figures 4A,B). Our results are consistent with previously published studies showing that GDF15 is necessary for the migration and invasion of high grade gliomas (18, 19) and strongly support the hypothesis that this molecule could be downstream of mechanical compression acting as a stress sensor that reacts to cytoskeletal and morphological changes (15) being critically involved in the mechanically-induced brain tumor progression.

### Screening for Identification of Compression-Induced Signal Transduction Mechanisms in Brain Cancer Cells

**Figure 7:**
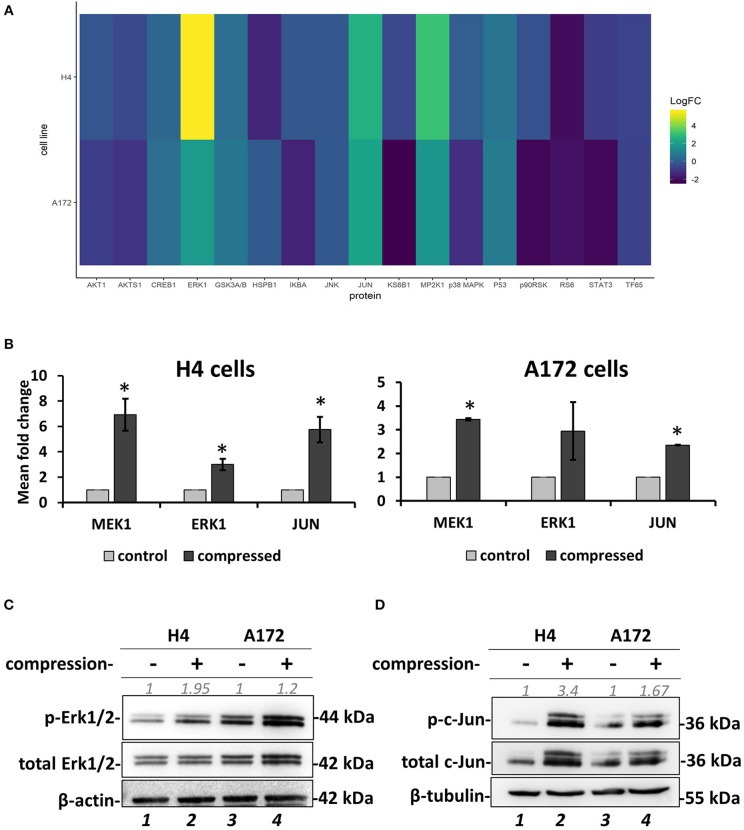
Screening for identification of compression-induced signal transduction mechanisms in brain cancer cells. **(A)** The heatmap depicts the change of Median Fluorescent Intensity (MFI) for the compressed cells at 16 h normalized to the MFI for the uncompressed cells of a representative experiment. **(B)** Quantification of the phosphoprotein analysis data for p-MEK1, p-Erk1, and p-Jun in compressed H4 and A172 using uncompressed cells as the control sample (3 biological replicates were performed; number of total replicates *n* = 3). **(C,D)** Validation of Erk1 **(C)** and c-Jun **(D)** activation using anti-phospho-p44/42 MAPK (Erk1/2) (Thr202/Tyr204), anti-p44/42 MAPK (Erk1/2), anti-phospho-c-Jun (S63), and anti-c-Jun in compressed H4 and A172 cells. Protein expression was quantified using ImageJ software and are indicated in gray font. The asterisk denotes a statistically significant difference compared to the control.

To investigate the mechano-transduction mechanism by which the compressive stress is transmitted into the cell nucleus to regulate gene expression and eventually the migration of brain cancer cells, we applied mechanical compression on H4 and A172 cells for 16 h, and whole cell lysates were screened for the identification of activated signaling pathways by using Multiplex Assay designed to detect 18 influential phospho-proteins. Analysis of the Multiplex Assay findings showed that Dual specificity mitogen-activated protein kinase kinase 1 (MAP2K1 or MEK1) was strongly activated by mechanical compression in H4 cells and to a less extent in A172 cells, as indicated by the phosphorylation level of MEK1 (S217/221 residue) in both cell lines (Figures 7A,B). Among all phosphoproteins tested in this screen, Mitogen-activated protein kinase 3 (MAPK3 or Erk1), which is a directly downstream substrate of MEK1, was found to be activated (T202/Y204 residue) in compressed cells (Figures 7A,B), while the transcription factor Activator Protein 1 (AP-1 or c-Jun), a transcription factor regulated by MEK/ERK pathway (45, 46), was also shown to be activated (S63 residue) in both cells lines. These results suggest for the first time a possible transduction mechanism of compression through MEK1/Erk1/c-Jun signaling axis in brain cancer cells, being more evident in the less aggressive H4 cells and to a less extent in A172 GBM cells, as indicated through a validation of the phospho-protein hits by Western Blotting (Figures 7C,D).

### MEK1/Erk1 Activation Is Necessary for Compression-Induced Signal Transduction and Regulation of Brain Cancer Cell Migration

**Figure 8:**
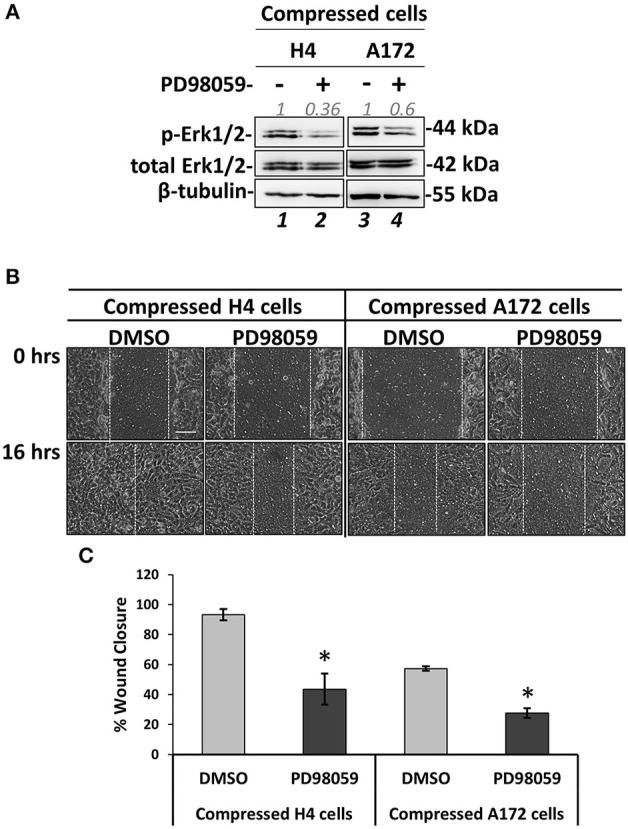
MEK1/ERK1 activation is necessary for compression-induced signal transduction and regulation of brain cancer cell migration. **(A)** Representative western blotting showing phosphorylated Erk1 (T202/Y204) and total Erk1 levels in compressed H4 and A172 treated with 20 μM PD98059 or DMSO. **(B)** PD98059-treated H4 and A172 cells were compressed by 4.0 mmHg in low-serum medium and then subjected to a scratch wound healing assay for 16 h. Control cells were treated with DMSO. Scale bar: 0.1 mm. **(C)** Graph showing the percentage wound closure as quantified using ImageJ software. Statistically significant difference in wound closure of PD98059-treated H4 and A172 cells compared to DMSO-treated cells is indicated with an asterisk (*) (2 different fields/condition; 3 independent experiments; number of total replicates *n* = 6; *p* < 0.05).

MEK1/Erk1 pathway is a well-characterized signaling pathway known to play a crucial role in cell survival and inhibition of apoptosis (47), while several studies have also shown that this pathway can be responsible for cancer cell migration and invasion (46–50). Nevertheless, the involvement of the MEK1 pathway in stress-induced brain cancer cell metastasis has not been described yet. To that regard, we employed an inhibitor of MEK1 (PD98059) that has been previously used to inhibit MEK1 activation *in vitro* and *in vivo* (51–55). We applied mechanical compression on H4 and A172 cells treated with PD98059 and we first confirmed that MEK1 phosphorylation was successfully inhibited in both cell lines by examining the levels of Erk1 phosphorylation, which is the downstream target of MEK1 (Figure 8A). Interestingly, the activation of c-Jun was not affected by MEK1/Erk1 inhibition, suggesting that this molecule could be independently regulated in compressed brain cancer cells (Supplementary Figure 5). Moreover, we observed that the migratory ability of both compressed cell lines was blocked (Figures 8B,C), while in the absence of mechanical compression MEK1/Erk1 was shown to be necessary only for the migration of A172 cells (Supplementary Figures 4C,D). These results suggest a critical involvement of MEK1/Erk1 signaling pathway in the compression-induced brain cancer cell migration, and especially in the migratory switch of the less aggressive H4 cells.

### GDF15 Regulation by MEK1/Erk1 Pathway and the Negative Feedback Loop to MEK1/Erk1 Activation in Compressed Brain Cancer Cells

**Figure 9:**
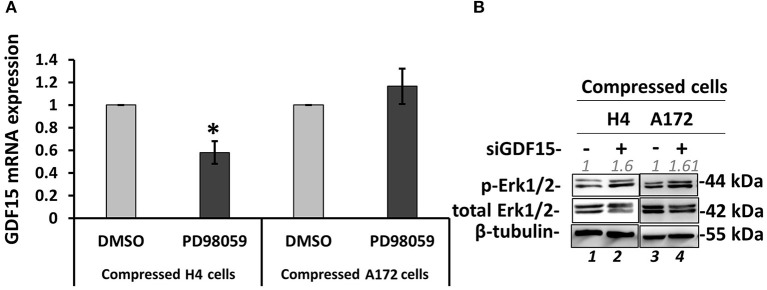
GDF15 regulation by MEK1/Erk1 pathway and the negative feedback loop to MEK1/Erk1 activation. **(A)** H4 and A172 brain cancer cells were pre-treated with 20 μM PD98059 or DMSO and were compressed by 4.0 mmHg in 2% FBS containing DMEM following by total RNA and protein isolation. GDF15 mRNA expression was quantified by qPCR. Each bar indicates the mean fold change ±SE of three independent experiments (number of total replicates *n* = 9). Asterisk (*) indicates a statistically significant difference (*p* < 0.05). **(B)** Western Blotting showing that Erk1 is activated upon GDF15 knockdown, as indicated by Erk1/2 phosphorylation levels (T202/Y204) in compressed siGDF15-treated H4 and A172 brain cancer cells. B-tubulin has been used to verify equal protein loading. Protein expression was quantified using ImageJ software and the fold change is indicated in gray font.

Several studies have shown that GDF15 can induce PI3K/Akt and Erk1/2 activation in esophageal squamous cell carcinomas, breast, cervical, gastric and prostate cancers (56–59), while regarding brain tumors it was found that an overexpression of GDF15 in brain cancer cells did not affected Erk1/2 activation (60). Since the relationship between GDF15 and Erk1/2 activation in brain cancer cells, and especially how it is regulated under compression, is not yet fully understood (60), we examined if GDF15 expression is affected by MEK1/Erk1 inhibition and if Erk1 activation is affected in compressed siGDF15-treated brain cancer cells. As shown in Figure 9, we observed a decrease in GDF15 mRNA levels in PD98059-treated compressed H4 cells, while its expression in the respective A172 cells was not affected (Figure 9A). Furthermore, Erk1 was found to be activated in compressed siGDF15-treated brain cancer cells (Figure 9B), suggesting that GDF15 could act as a negative regulator of Erk1 activation in response to compression. Our results indicate for the first time that GDF15 could be regulated by MEK1/Erk1 signaling pathway, at least in the less aggressive brain cancer cells, while it could also act as a negative regulator of MEK1/Erk1 activation in order to regulate its expression levels in compressed brain cancer cells.

## Discussion

Mechanical forces developed in the tumor microenvironment and matrix stiffness are recently suggested to be two distinct biomechanical abnormalities developed in the tumor microenvironment that can independently affect tumor progression (42, 61, 62). However, while the effect of matrix stiffness on tumor progression has been studied extensively, the effect of mechanical forces on cancer cell behavior and especially on brain cancer cell migration remain elusive. Thus, in order to examine the role of compressive forces in brain tumor progression, and especially in brain cancer cell migration, *in vitro*, we first examined their effect on MCS composed of two brain cancer cell lines with distinct metastatic potential embedded in an agarose matrix. According to previous studies, the agarose matrix is supposed to mimic the confinement of the normal host tissue that resists to tumor expansion, resulting in the development of compressive forces in the tumor interior in the absence of any additional stiffness-induced effect caused by the presence of a collagen matrix (6, 38, 40). As previously suggested for breast and colon tumor spheroids growth (6, 38, 40), we observed that the presence of the surrounding matrix impaired the growth of brain MCS as compared to spheroids grown in free suspension. Interestingly, this effect was in accordance with the effect caused on the growth of glioblastoma tumor spheroids embedded in a collagen matrix (41), which is supposed to create confined conditions to tumor spheroids similar to the effect caused by the agarose gel. With the use of computational analysis, we further estimated that the level of the resultant compressive stress in MCS was in the range of 0–60 mmHg (0–8 kPa), which is in agreement with previously established *in vitro* and *in vivo* studies (6, 24, 38, 42, 43).

Next, by using our established transmembrane pressure device for the compression of cell monolayers, we found that compression can differentially regulate the migration of brain cancer cells. More specifically, mechanical compression induced the migration and decreased the proliferation of the non-metastatic H4 cell line, while a less pronounced effect was observed for the highly aggressive A172 cells. This effect could possibly be explained by the fact that A172 cells are derived from high grade gliomas where the presence of compressive forces is more pronounced, suggesting that these cells are less responsive because they are previously exposed to mechanical compression. Moreover, although Kaufman et al. suggested that a stiffer collagen matrix, that is supposed to increase the compressive forces applied on cells, promotes the invasion of the highly aggressive U87 glioblastoma cell line (41), a stiffer matrix does not always means higher levels of mechanical compression, as the cells can continuously remodel the collagen fibres in order to invade into the surrounding matrix. This cell-matrix interaction might change the mechanical compression applied on cells and thus, our results support the recent hypothesis that matrix stiffness and mechanical forces are two distinct biomechanical abnormalities that can independently regulate tumor progression and thus, their effect should be studied separately (42, 62). By further analyzing the gene expression of these cells, emphasizing on genes related to actin cytoskeleton and migration, we observed that the expression of RhoB GTPase and Rac-1 was differentially regulated in compressed cells, with RhoB changes being more evident, showing a strong increase in H4 cells and a slight decrease in A172 cells. Regarding the other small GTPases, RhoA and cdc42 mRNA expression was slightly increased in compressed A172 cells, exhibiting negligible changes in compressed H4 cells. It is of note that Rho A/B/C GTPases can get activated by growth and stress stimuli and are important regulators of cell and tissue morphology and function, acting mainly through actin cytoskeleton reorganization (17). In particular, RhoB has been found to be expressed in high-grade glioma, while depletion of this molecule impaired proliferation and survival of GBM cells through a STAT3-dependent regulation of p53 and p21 expression, and that knockdown of RhoB found to impair the *in vivo* tumorigenic potential of GBM cells (16). However, when RhoB was silenced in compressed H4 and A172 cells did not affect their migratory ability, letting us to hypothesize that this protein might be subjected to posttranslational modifications (e.g., farnesylated or geranylgeranylated RhoB) to regulate cell migration and actin cytoskeleton organization (63) or other members of the small GTPases family, such as RhoA/C, Rac-1, or cdc42, could be activated in response to compression in order to rescue RhoB silencing. Our results support that there is a dynamic interplay among all small GTPases in order to organize actin cytoskeleton and regulate brain cancer cell migration under compression, and thus further investigation is needed to identify their exact role in this process. Along with small GTPases, we also found that GDF15 was dramatically upregulated in response to compression in both cell lines. Although it was expected that upregulation of GDF15 is directly linked to higher migration rates, we observed that while GDF15 was upregulated in compressed A172 cells, their migratory ability was unaffected or tend to be decreased by mechanical compression without a significant difference compared to control cells. This effect could be easily explained by the fact that GDF15 expression is higher in highly aggressive brain cancer cells (19), and by combining this with the fact that mechanical compression is more pronounced in high grade gliomas, we could explain why a further upregulation of this molecule could not have an additional effect on the migration of these cells. However, as it is already suggested, GDF15 is strongly regulated by morphological changes and cytoskeleton disruption (15). Thus, it could be elevated in both cell lines due to a disruption of actin cytoskeleton caused by the applied mechanical compression, being necessary for actin cytoskeleton re-organization that could indirectly and in combination with other molecules, promote cell migration. This is further supported by the fact that GDF15 is necessary for the migration of both cell lines, as indicated by the decreased migratory ability of compressed cells knockdown for GDF15.

Finally, by a phospho-proteomic screen we analyzed the possible signaling pathways that could get activated by mechanical compression. We found a strong activation of MEK1/Erk1 pathway in the compressed H4 cells (about 7-fold change) and to a less extent in compressed A172 cells (3-fold change). Interestingly, 45–57% of GBM cases tested, showed activating mutations in the *EGFR* gene, which is one of the main activators of Ras/Raf/MEK1/Erk1/2 signaling axis and it is found to be involved in proliferation, migration and invasion of brain cancer cells (1, 64, 65). Moreover, as already mentioned, the level of mechanical compression is higher in high grade gliomas and this could also be a novel explanation for the increased MEK1/Erk1 activation in those tumors. Based on the fact that A172 cells are derived from GBM, we could hypothesize that MEK1/Erk1 pathway is pre-activated in these cells, which can subsequently cause a less pronounced compression-induced migratory effect.

Several studies have shown that GDF15 can induce PI3K/Akt and Erk1/2 activation in esophageal squamous cell carcinomas, breast, cervical, gastric, and prostate cancers (56–59), while in brain tumors it was only found that an overexpression of GDF15 did not affected Erk1/2 activation (60). However, our results showed that Erk1 is activated when GDF15 has been knockdown, suggesting a possible negative feedback loop between GDF15 and Erk1 activation in brain cancer cells under compression. Moreover, we observed a decrease in GDF15 mRNA levels upon MEK1/Erk1 inhibition in compressed H4 cells, suggesting that this pathway could get activated by compression in order to regulate GDF15 expression. It should be also noted that in both cases, the migratory ability of both cell lines was inhibited, suggesting that MEK1/Erk1 activation and GDF15 expression can synergistically regulate brain cancer cell migration under mechanical compression. However, the fact that MEK1/Erk1 pathway and GDF15 expression are also necessary for the migration of uncompressed A172 cells but not for that of uncompressed H4 cells, suggest for the first time that the presence of compressive forces in brain tumors can *de novo* activate MEK1/Erk1 pathway to promote the aggressiveness of brain cancer cells rendering this pathway along with GDF15 molecule as potential targets for the compression-induced brain tumor progression.

**Figure 10:**
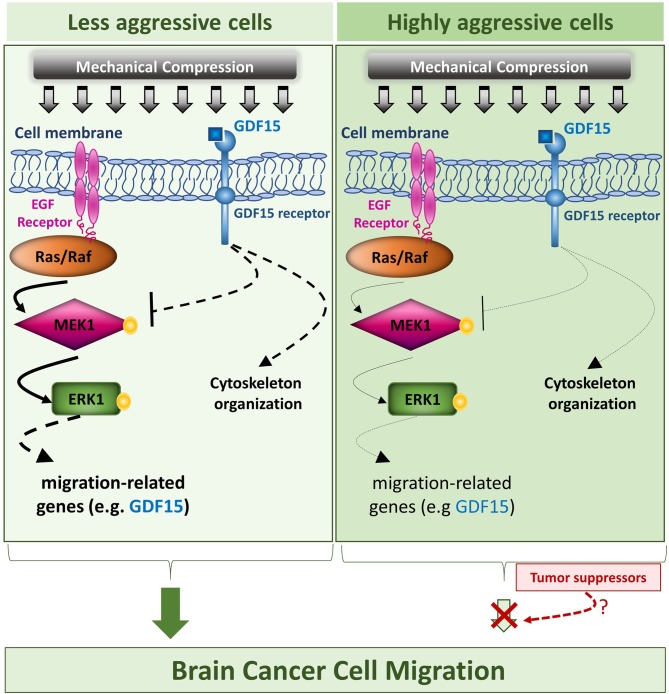
Proposed mechanism of this study. The proposed mechanism of the present study is that compressive force, applied on brain cancer cells, could activate MEK1/Erk1 pathway through EGFR/Ras/Raf activation, which as it is widely known, is just upstream of MEK1. Subsequently, MEK1/Erk1 could regulate the expression of several migration-related genes including GDF15, in order to re-organize the compression-disrupted actin cytoskeleton, which eventually facilitates cell migration. Finally, through a possible negative feedback loop, GDF15 could bind to its receptor and then suppress MEK1/Erk1 activation in order to regulate its levels in compressed cells. In this diagram, the arrows are of different width, so that thick arrows indicate a stronger compression-induced effect in the less aggressive cells, while thin arrows indicate a weaker effect in the highly aggressive cells, which could permit a possible tumor suppressing pathway to hinder the compression-induced MEK1/Erk1 mediated migratory effect.

Collectively, our results suggest that mechanical compression applied on brain cancer cells could activate MEK1/Erk1 pathway through EGFR/Ras/Raf activation, which as it is widely known, is just upstream of MEK1. Subsequently, MEK1/Erk1 could regulate the expression of several migration-related genes including GDF15, in order to re-organize the compression-disrupted actin cytoskeleton, which eventually facilitates cell migration. Through a possible negative feedback loop, GDF15 could bind to its receptor and then suppress MEK1/Erk1 activation in order to regulate its levels in compressed cells. Finally, as indicated in Figure 10, the fact that mechanical compression has a weaker effect in the highly aggressive cells could permit a possible tumor suppressing pathway to hinder the compression-induced MEK1/ERK1 mediated migratory effect. Although many questions still remain regarding the comprehensive mechanism involved in compression-induced brain tumor progression, including whether Erk1 can directly regulate GDF15 expression, how exactly the small GTPases could be regulated by compressive stress and what is the exact role of GDF15 in brain cancer cell migration and cytoskeleton re-organization under compression, this is actually the first study connecting compression-induced migratory profile of brain cancer cells with MEK1/Erk1 activation, small GTPases and GDF15 expression regulation, rendering them as potential targets for future anti-metastatic therapeutic innovations to treat brain tumors.

## Data Availability Statement

The raw data supporting the conclusions of this manuscript will be made available by the authors, without undue reservation, to any qualified researcher.

## Author Contributions

MK was responsible for designing the study, performing the experiments, analyzing data, interpreting results, and writing the paper. CV performed mechanical testing experiments and mathematical modeling. AM and VP performed the phosphoproteomics experiments. CF analyzed data of phosphoproteomics experiments. LA supervised phosphoproteomics experiments and data analysis. TS was responsible for designing the study, analyzing data, interpreting results, and editing the manuscript. All authors have read and approved the final manuscript.

### Conflict of Interest

AM, VP, and LA were employed by company “ProtATonce Ltd.” The remaining authors declare that the research was conducted in the absence of any commercial or financial relationships that could be construed as a potential conflict of interest.
