# Supplementary material for: Mechanical Compression Regulates Brain Cancer Cell Migration Through MEK1/Erk1 Pathway Activation and GDF15 Expression
Source: Front Oncol. 2019 Sep 27;9:992. doi: 10.3389/fonc.2019.00992 (PMC6777415; doi:10.3389/fonc.2019.00992)

Supplementary Material

# Supplementary Methods

**Phosphoproteomics**. 18 capture antibodies coupled to Luminex magnetic beads and 18 biotinylated detection antibodies were multiplexed to create the bead mix and the detection mix, respectively. 50ul of the coupled beads (bead mix) were incubated with the samples on a flat bottom 96-well plate on a shaker at 900 rpm for 90 minutes at room temperature. Then, detection mix was added, and the samples incubated on a shaker at 900rpm for 60 minutes at room temperature. The final step was the addition of freshly prepared SAPE solution (Streptavidin, R-Phycoerythrin conjugate, Cat Nr: S866, Invitrogen) for the detection of the signal. 15 minutes after the incubation with SAPE, samples were measured with the Luminex FlexMAP 3D instrument. The following phospho-proteins were measured: mitogen-activated protein kinase 3 (ERK1, Cat Nr: P-MK03-A01), Dual specificity mitogen-activated protein kinase 1 (MEK1, Cat Nr: P-MP2K1-A01), Mitogen-activated protein kinase 12 (p38, Cat Nr: P-MK12-A01), RAC-alpha serine/threonine-protein kinase (AKT1, Cat Nr: P-AKT1-01), Signal transducer and activator of transcription 3 (STAT3, Cat Nr: P-STAT3-A01), Heat shock protein beta-1 (HSP27, Cat Nr: P-HSPB1-A01), Signal transducer and activator of transcription 5A (STAT5, Cat Nr: -P-STAT5-A01), Cellular tumor antigen p53 (p53, Cat Nr: P-P53-A01), Glycogen synthase kinase-3 alpha/beta (GSK3A/B, Cat Nr: P-GSK3A/B-A01), Mitogen-activated protein kinase 9 (JNK, Cat Nr: P-MK09-A01), 40S ribosomal protein S6 (RS6, Cat Nr: P-RS6-A01), Ribosomal protein S6 kinase beta-1 (p70S6K, Cat Nr: p-KS6B1-A01), Ribosomal protein S6 kinase alpha-1 (RSK1, Cat Nr: P-KS6A1-A01), Cyclic AMP-responsive element-binding protein 1 (CREB1, Cat Nr: P-CREB1-A01), NF-kappa-B inhibitor alpha (Ik-Ba, Cat Nr: P-Ik-Ba-A01), Proline-rich AKT1 substrate 1 (AKTS1, Cat Nr: P-AKTS1-A01), Transcription factor p65 (TF65, Cat Nr: P-NFkB-A01), Transcription factor AP-1 (JUN, Cat Nr: P-JUN-A01)

**Normalization of phosphoproteomics measurements.**

*Limit of detection filtering*

For each of the 18 phosphoproteomic measurements a limit of detection was calculated according to the following formula,

$lod_{i}=\mu_{blank,i}+1.65\sigma_{blank,i}$ *(1)*

Where, $lod_{i}$ is the limit of detection for each protein $i$, while $\mu_{blank,i}$ and $\sigma_{blank,i}$ are the mean and standard deviation of the triplicate blanks, for each protein, respectively.

Using the values from equation *(1)* only the phosphoproteins that had a measurement higher that the limit of detection was kept. This filtering approach resulted in the exclusion of STAT5 from further visualization and analysis.

*Phosphoprotein MFI normalization*

The MFI values for each protein were normalized according to the following formula,

$MFI_{normalized i,j}=2 \frac{MFI_{treated i,j}-MFI_{control i}}{\frac{MFI_{treated i,j}+MFI_{control i}}{2}}$ *(2)*

Where, $MFI_{treated i,j}$is the mean fluorescent intensity of the *i-th* phosphoprotein at the *j-th* time point for the compressed cells and $MFI_{control i}$ is the mean fluorescent intensity for the *i-th* phosphoprotein for the uncompressed cells.

This normalization procedure was chosen instead of the most commonly used Fold change in order to better visualize the change in MFI for some proteins with low basal MFI (e.g AKT1).

# Supplementary Figures and Tables

## Supplementary Figures


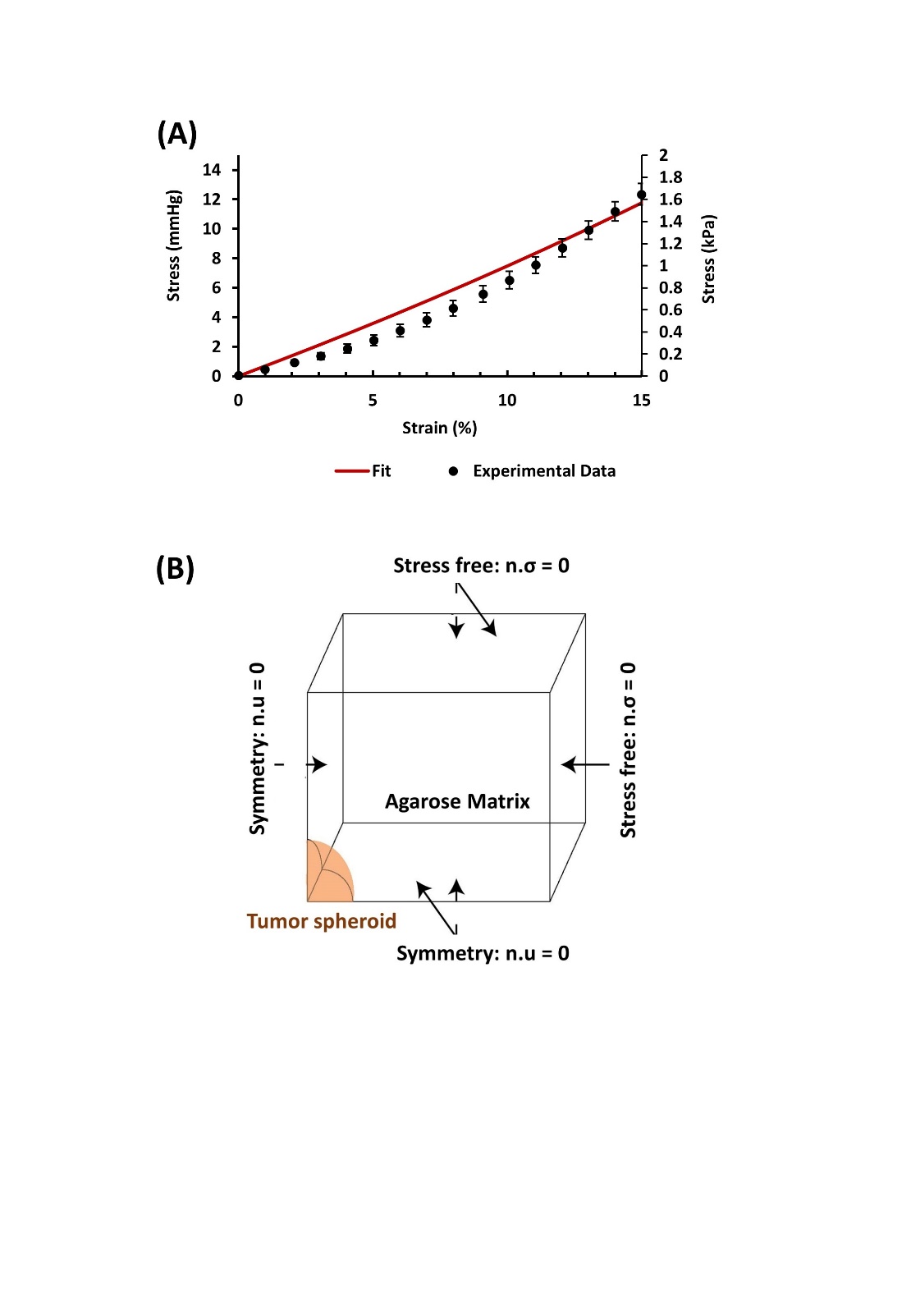


**Supplementary Figure 1. (A)** Representative fitting of the neo-Hookean equation to the experimentally measured stress-strain response of 1% agarose gel**. (B)** A tumor cell spheroid grown within an agarose matrix was modelled (orange color). Due to symmetry the one eight of the domains was solved applying a symmetry boundary condition at the symmetric boundaries and a stress-free condition at the free surfaces, where **n** is the unit normal vector and **u** is the displacement vector. The continuity of the displacements and the normal stress at the spheroid-matrix interface is implemented automatically by the software.


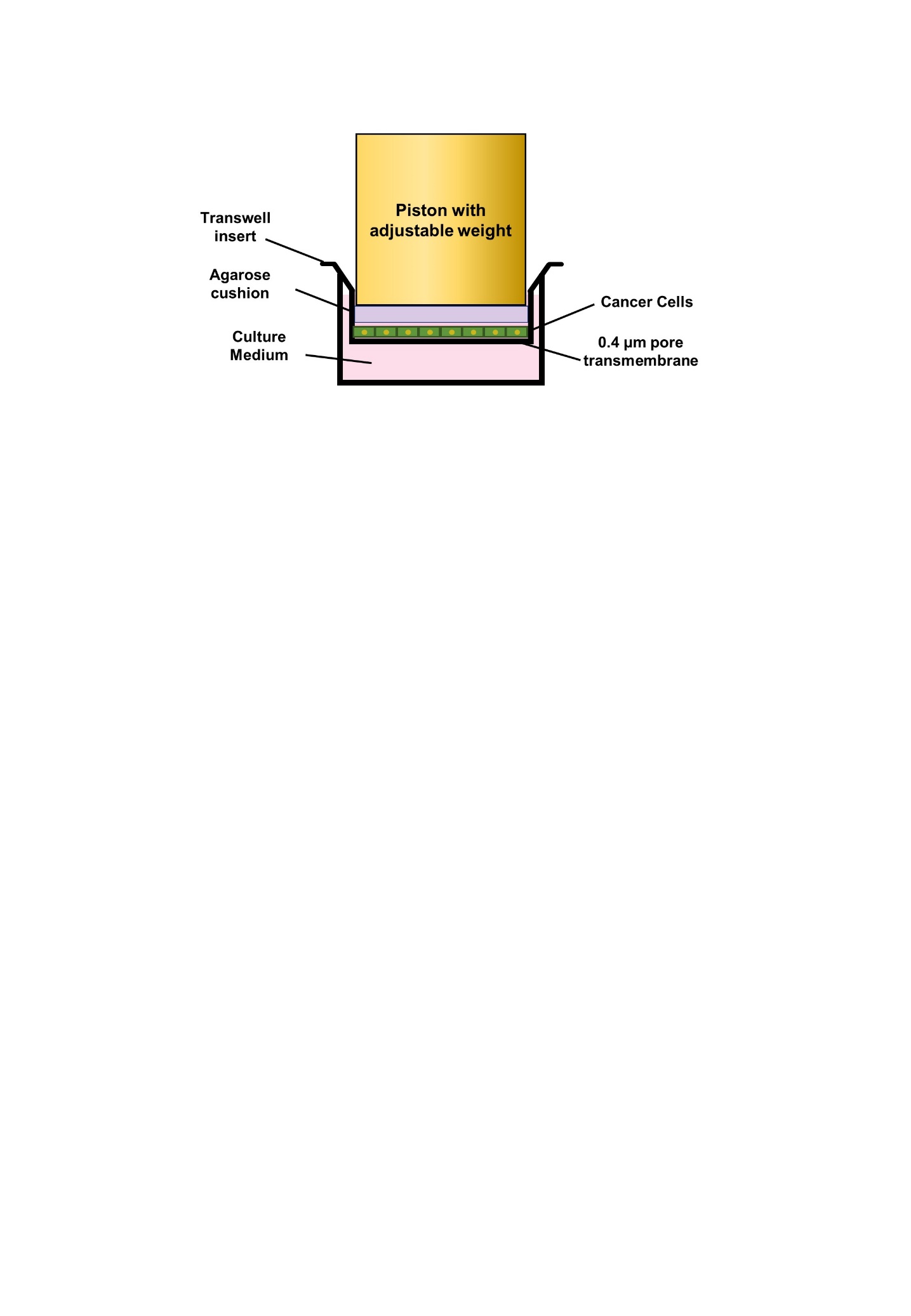


**Supplementary Figure 2. A schematic of the transmembrane pressure device.** Brain cancer cells were grown to form a monolayer on the transmembrane of a 0.4 μm transwell insert and a piston of adjustable weight applied a predefined compressive stress. Control cells were covered with an agarose cushion only. Τhe picture was reproduced with permission from ref.


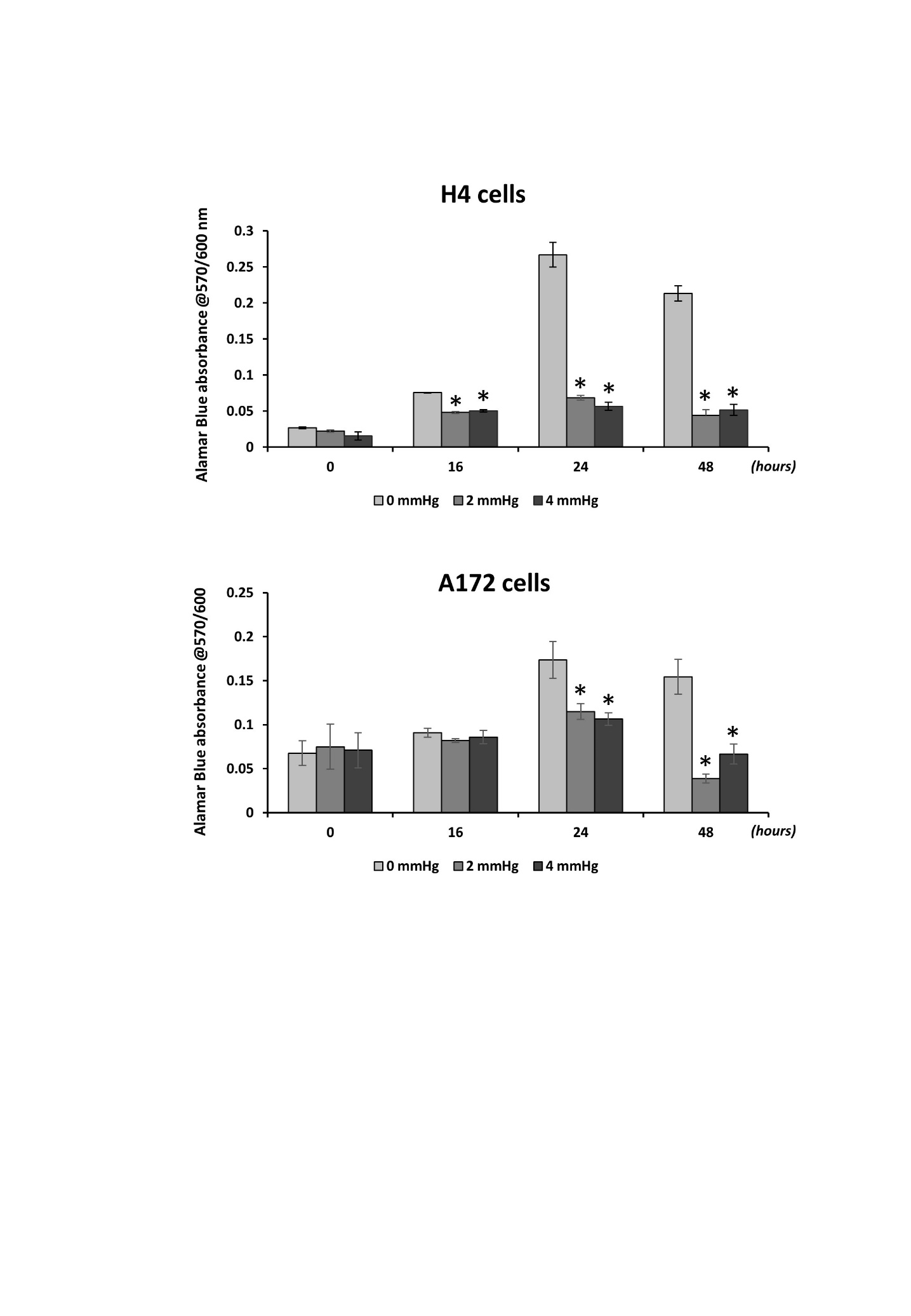
**Supplementary Figure 3. Cell viability assay of compressed and control brain cancer cells for 48 hours.** Brain cancer cells lines (H4 top, A172 bottom) were counted and seeded with equal density in 6-well transwell inserts. Alamar Blue was added in culture medium (10%) and absorbance was measured at 0, 16, 24 and 48 hours of compression (4.0 mmHg) at 570/600 nm. Absorbance of Alamar Blue is indicative of the total cell number.


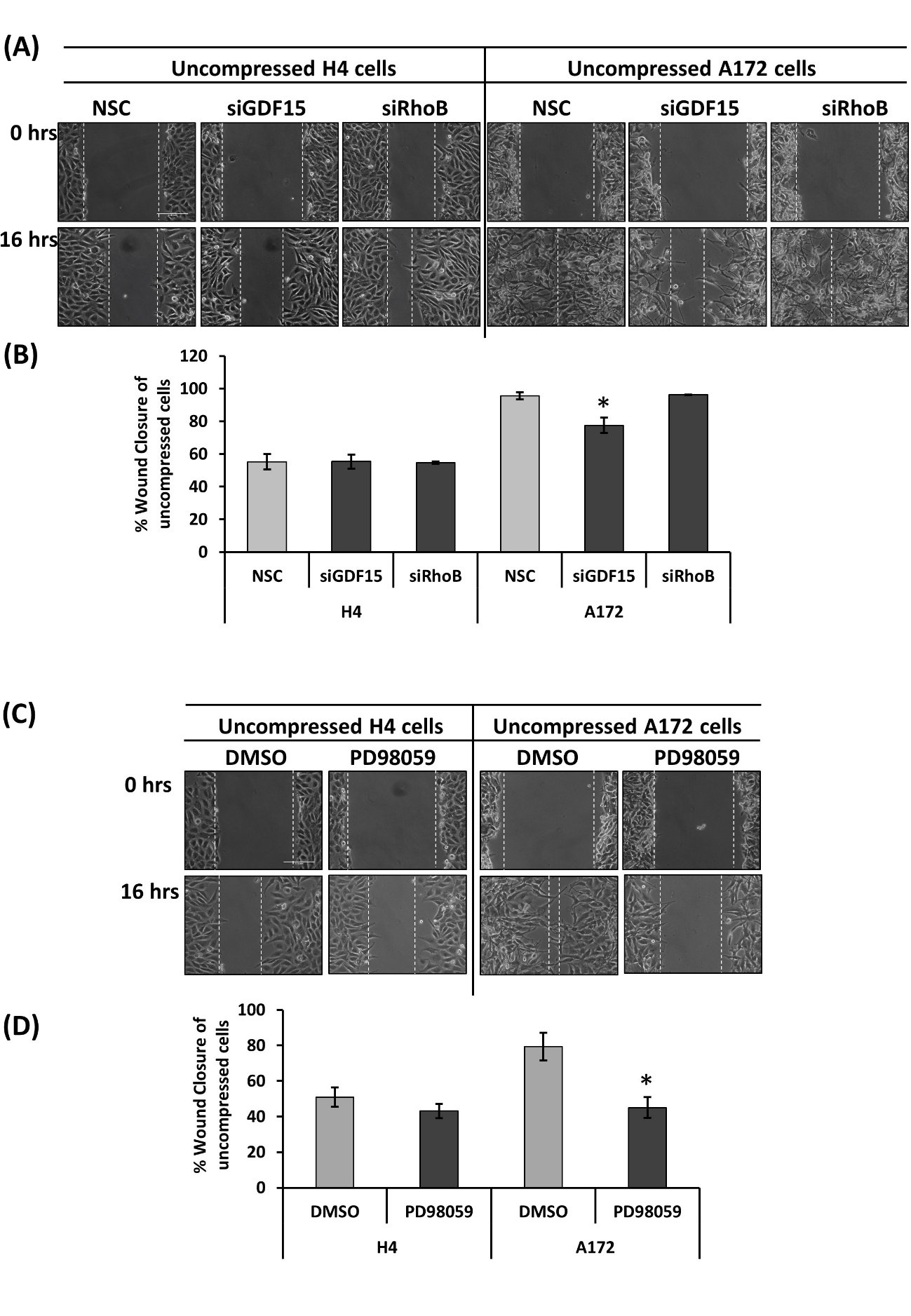
**Supplementary Figure 4. Effect of siGDF15, siRhoB and PD98059 treatment on brain cancer cell migration in uncompressed conditions. (A**) Uncompressed H4 and A172 cells were treated with control siRNA (NSC) or siRNA against GDF15 (siGDF15) or RhoB (siRhoB) for 48 hours and then were subjected to a wound healing assay for 16 hours. Bar graph represents the average of %Wound Closure for each condition as quantified using the ImageJ software. **(B)** Uncompressed H4 and A172 cells were pre-treated with PD98059 or equal volume of DMSO and then subjected to a wound healing assay for 16 hours. Bar graph represents the average % Wound Closure as quantified using the ImageJ software. Scale bar 0.15mm.


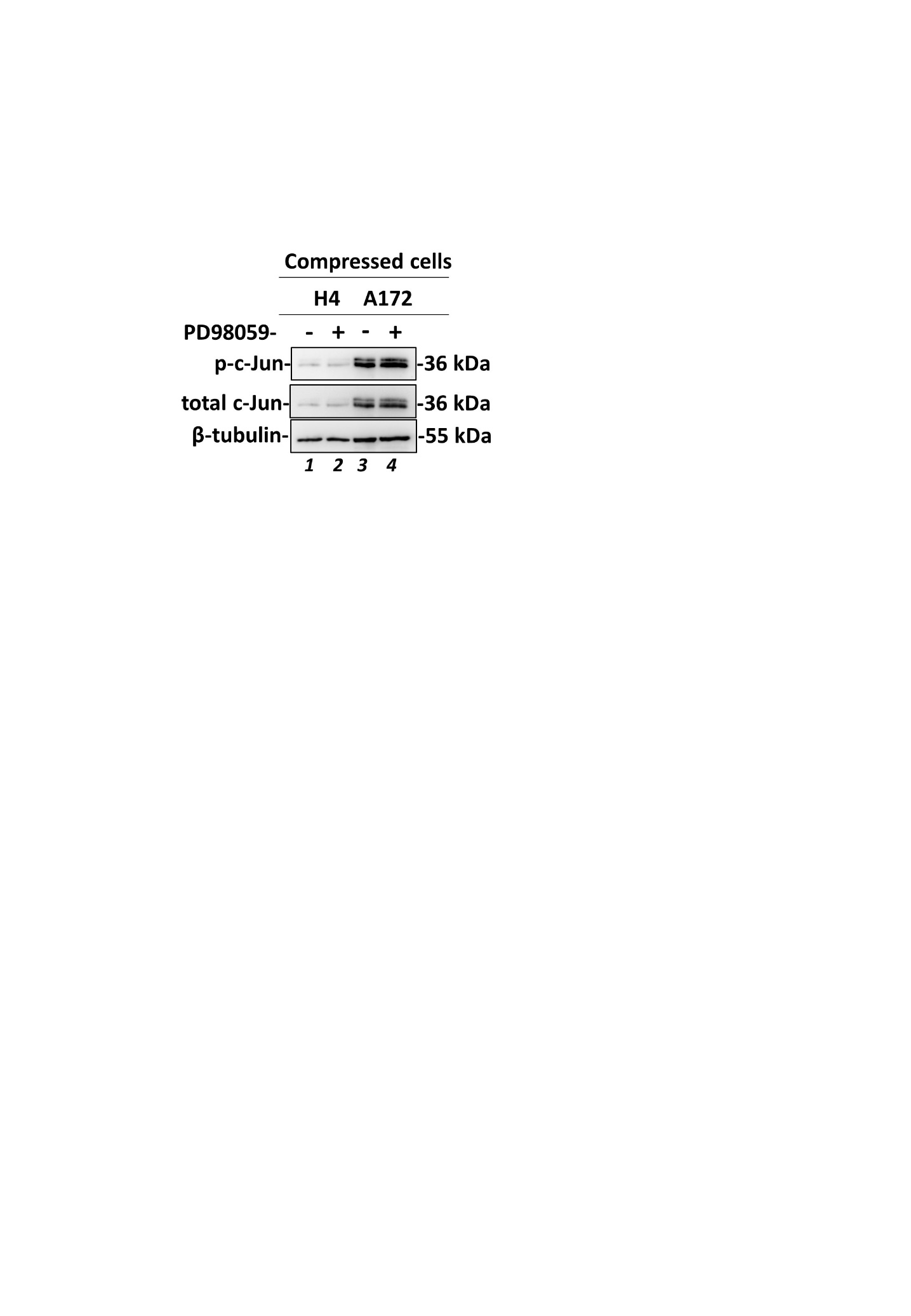


**Supplementary Figure 5. MEK1 inhibition did not affect c-Jun activation in compressed brain cancer cells**. Western Blotting showing c-Jun activation using anti-phospho-c-Jun (S63) and anti-c-Jun total in compressed H4 and A172 cells treated with 20μΜ PD98059 or DMSO. B-tubulin was used to verify equal protein loading.

## Supplementary Tables


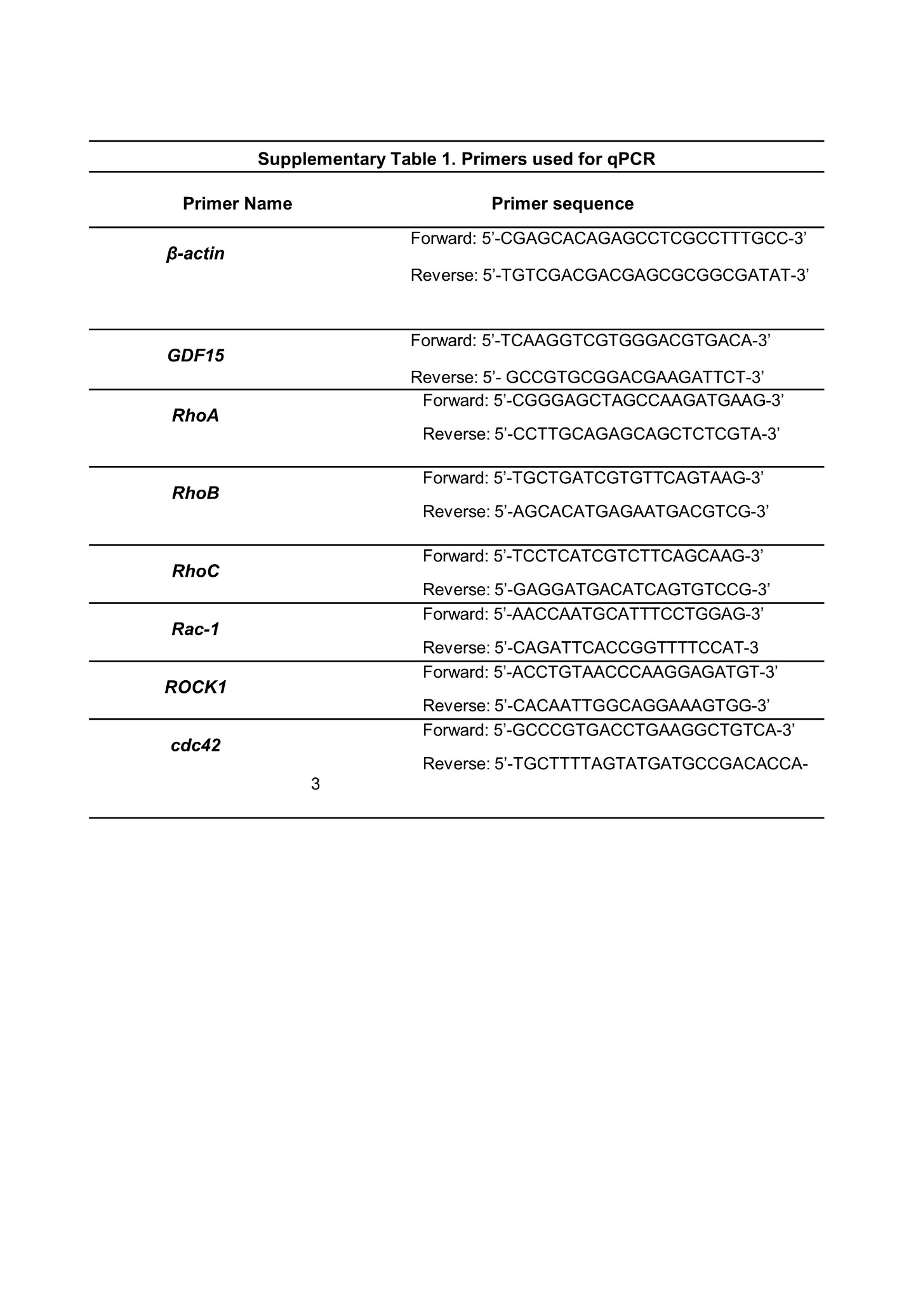

Supplement: Supplementary file 1 [file Data_Sheet_1.docx]
